# Comparison of Oncological and Surgical Outcomes Between Formal Pancreatic Resections and Parenchyma-Sparing Resections for Small PanNETs (<2 cm): Pancreas2000 Research and Educational Program (Course 9) Study Protocol

**DOI:** 10.3389/fmed.2020.00559

**Published:** 2020-09-10

**Authors:** Antonio Pea, Lulu Tanno, Taina Nykänen, Pooja Prasad, Ceren Tunçer, Stuart Robinson, Giovanni Marchegiani

**Affiliations:** ^1^Department of Surgery, Istituto Pancreas, Ospedale Universitario Integrato Verona, Verona, Italy; ^2^Department of General Surgery, University Hospital Southampton NHS Foundation Trust, Southampton, United Kingdom; ^3^Department of Surgery, Hyvinaa Hospital, Hyvinkaa, Finland; ^4^Freeman Hospital, Newcastle upon Tyne, United Kingdom; ^5^School of Medicine, Koc University Research Centre for Translational Medicine, Istanbul, Turkey

**Keywords:** pancreatic neurendocrine tumor, parenchyma sparing pancreatectomy, pancreatic resection, oncological outcomes, survival

## Abstract

Pancreatic neuroendocrine tumors (PanNETs) are rare tumors but incidence is increasing. An increasing number of these tumors are diagnosed incidentally when they are small (<2 cm) and when patients are asymptomatic. The European Neuroendocrine Tumor Society (ENETS) recommends conservative watch and wait policy for these patients. However, best surgical approach (parenchyma-sparing or formal oncological resection) for these small tumors when surgery is indicated is currently unknown. Parenchyma-sparing resections such as enucleation is associated with higher risk of post-operative morbidity compared to formal oncological resections. They are also be associated with potentially inadequate surgical margin clearance and with lack of lymphadenectomy for full pathological staging.

**Method:** This study is a retrospective study and the aim is to analyze pre-operative clinical predictors of nodal metastases for small PanNETs to identify which patients are at a lower risk of lymph node metastases and are therefore suitable for parenchyma-sparing resection.

**Conclusion:** The primary endpoint of this study is to determine if pre-operative clinical predictors such as tumor size are associated with lymph node involvement in small PanNETs.

## Background and Rationale for the Study

Pancreatic neuroendocrine tumors (PanNETs) are considered rare neoplasms with a incidence of 0.8 per 100,000 individuals (1). Autopsy studies have highlighted a prevalence ranging from 1 to 10% in the general population, suggesting that PanNETs are not always symptomatic leading to clinical diagnosis (2, 3). In recent years, an increasing number of small and asymptomatic PanNETs are diagnosed incidentally on routine abdominal imaging. The risk for metastases has been associated with tumor size and grade based on high proliferative index (4, 5), periodic observation without resection has been advocated for small low grade tumors (6).

European Neuroendocrine Tumor Society (ENETS) guidelines suggests that in a selected patients with a small and asymptomatic PanNETs (6). A small number of studies have demonstrated a conservative watchful imaging-based management is safe in the short-term (7–9). However, these small prospective studies are limited by relatively short follow-up (median 45 months). Hence, ENETS is currently conducting a study on Asymptomatic Small Pancreatic Endocrine Neoplasms (ASPEN) to evaluate the most appropriate management for these patients (NCT 03084770). Surgical resection is only recommended in young and healthy patients due to the absence of available data on long-term follow-up (6). In this regard, parenchyma- sparing resections have been performed for small tumors harboring a negligible risk on lymph node metastases and, according to the ENETS guidelines, are now proposed to selected patients affected by small PanNETs when conservative management is contraindicated (e.g., young patients or patients who refuse observational management).

The main oncological limitations of these techniques are the risk of inadequate surgical margin clearance and the absence of lymphadenectomy. Parenchyma spearing techniques are indeed characterized by a higher risk of postoperative morbidity than formal resections, but with a lower risk of long-term exocrine and endocrine pancreatic insufficiency (7, 10). For example, when considering enucleation, technical contraindications include tumors in close proximity to the main pancreatic duct as this is associated with a high risk of pancreatic fistula. Therefore, oncological and technical factors have to be evaluated when considering parenchyma-spearing resection for sporadic small PanNETs.

## Objectives and Outcome Measures

### Primary Objective

This study aims to analyze preoperative clinical predictors of nodal metastases for small PanNETs to understand which patients are at a lower risk of lymph node metastases and therefore are suitable for parenchyma-spearing resection.

### Secondary Objectives

- Comparison of pathological and surgical outcomes (complication rates, length of stay) of PanNETs treated with parenchyma spearing resections to tumors treated with formal oncological resections.
- To assess if there is any association between the type of resection patients receive and on disease-free and overall survival.

### Outcome Measures

The primary end-point of this study is to determine if pre-operative clinical predictors (presence of absence of pain, functionality of tumor, tumor size, tumor location, associated duct dilatation) are associated with lymph node involvement in small <2 cm PNETs. The secondary end-point is to compare pathological and surgical outcomes and survival with types of surgeries patients received.

## Study Design

The study is a retrospective multi-center cohort study. All data will be anonymized and stored in a multi-center database. Individual centers participating in this study are advised to register the study with the appropriate department within their institution. Ethical approval will be sought from the ethical board of each participating institution separately.

Patients that underwent surgery for well-differentiated PanNETs ≤ 2 cm will be included in the study.

### Inclusion Criteria

- Surgically resected well-differentiated PanNET
- Largest diameter ≤ 2 cm on histology
- Grade 1 and Grade 2 PanNETs based on ki67 (≤20%) or mitotic index ( ≤20/10 HPF).

### Exclusion Criteria

- Pre-operative chemotherapy
- Grade 3 disease based on Ki67 or mitotic index on histology report
- Poorly differentiated or neuroendocrine carcinoma on histology
- Confirmed metastatic disease at the time of diagnosis
- Follow up of <6 months will be excluded from the analysis.

## Study Data

A multinational multi-center database will be created to collect and collate data from the medical records of participating institutions.

### Recruitment

ENETS centers of excellence and other institutions which perform large volume pancreatic resections are invited to take part in the study. Allocated data support medical personnel appointed by the institutions to collect the data for this study will collect anonymized data on:

1. Demographic characteristics (age, sex), clinical presentation, types of diagnostic imaging, radiological variables including location, size, bile duct and main pancreatic duct dilatation, intraparenchymal or exophytic radiological pattern, preoperative Ki67 labeling index, somatostatin receptor imaging data will be included in the study.
2. Details of surgery, length of stay and associated complications such as pancreatic fistula, post-operative hemorrhage requiring intervention, return to theater
3. Pathological results including resection margin, tumor size, ki-67, total number of lymph node examined, and the number of positive lymph nodes, presence of vascular or perineural invasions.
4. Survival—if patients are alive, then the date of last follow up, if deceased then the date of death will be recorded.
5. Disease free survival—date of recurrence and the site of recurrence will be recorded and will be used to calculate disease free survival.

### Participant Identification

Each center will have different procedures. At University hospital Southampton, there is an existing database on all patients diagnosed with neuroendocrine tumors. Similar database would also exist for other European neuroendocrine tumor society accredited centers of excellence. This database would be used to identify patients.

People who would be involved in inputting the data would be those of services users already having access to the data or clinical member of the staff. However, there will be no patient identifiable information recorded in the database for the study.

## Statistics and Data Analysis

All the data analysis will be performed by the Pancreas 2000 project members, using IBM SPSS version 25 for windows.

### Sample Size

Due to the retrospective nature of the study, no formal sample size calculation has been performed. However, this study protocol have received significant interest from large number of international institutions, both within the UK and EU. We anticipate participation from around 20 centers internationally with a minimum inclusion of 20 patients per center.

### Demographics Analysis

Categorical data will be presented as proportions; continuous data will be presented as either mean (standard deviation) or median (interquartile range) as appropriate. Continuous variables will be analyzed using *t*-test if parametric or chi-squared test if categorical.

### Primary Endpoint Analysis

The association between clinical, surgical (intra-operative and post-operative) and lymph node involvement will be analyzed using correlations. If there is an association with a specific parameter and lymph node involvement, a receiver operative characteristic (ROC) analysis will be performed to identify the predictive cut-off value.

Patients with multiple endocrine neoplasia type 1 (MEN-1) will be analyzed separately.

### Secondary Endpoint Analysis

Disease-free survival and overall survival will be analyzed using the Kaplan-Meier method and the log-rank test. Different pathological and pre-clinical factors and their association with survival will be evaluated using univariate and multivariate analysis. A *p* < 0.05 will be considered statistically significant.

### Data Management

Data management plan for this project is publicly accessible from https://dmponline.dcc.ac.uk/. The document is titled **“Comparison of oncological and surgical outcomes between formal pancreatic resections and parenchyma-sparing resections for small PanNETs (<2 cm).”**

All data generated will be stored in a password protected University of Southampton iSolutions secure data storage service which is regularly backed up. The principle investigator and those of the pancreas 2000 study group members (those names listed above) will be the only people who will have access to the anonymized multi-center database. The data will be used for analysis and for publication. The anonymized data will be kept for 10 years by the principle investigator and destroyed after this time.

### Expected Results

This study has recruited around 800 patients from several centers already and aim to complete the analysis by the end of 2020. We have not performed any provisional analysis at this point in time, but we anticipate clinical parameters collected such as associated symptoms, site of tumor, associated duct dilation as well as tumor size maybe associated with lymph node metastasis. We hope that this will provide valuable additional parameters to aid in the management of these controversial and challenging group of patients.

## Dissemination Policy

### Authorship and Publication Policy

Authorships will be based on the recommendations from the international committee of medical journal editors (ICMJE). The first five authors will be the members of the Pancreas 2000 participants of this project (AP, TN, LT, PP, and CT). The last two authorship positions are reserved for the two mentees of this group (GM and SR). All other authors will be listed based on the number of cases provided. A minimum of 20 patients per center is required for authorship. Two authors will be listed as co-authors from participating institutions. If the institution contributes more than 40 patients to this study, additional co-authorships will be allocated. We ask each participating institution to submit their data to the lead coordinator of this project (antonio.pea@univr.it) by October 2020.

### Insurance

The necessary trial insurance is provided by the sponsor. University Hospital Southampton NHS trust holds standard NHS Hospital Indemnity and insurance cover with the NHS litigation Authority for NHS Trust in England, H4RT V1.1 3rd April 2017 which apply to this trial. However, the University Hospital Southampton is unable to act as a sponsor for non-UK sites and we advise that the individual sites seek their own indemnity and insurance.

## Ethics Statement

This study has obtained ethical approval from the research Ethics Committee in the United Kingdom (REC reference:20/LO/0201. Participating institutions outside of UK should seek ethical approval separately.

## Author Contributions

The study protocol was written jointly between AP, LT, TN, PP, and CT as part of the pancreas2000 study group. LT and AP was involved with editing the protocol based on the feedback and comments given by SR and GM who are the mentors of the group. All authors contributed to the article and approved the submitted version.

## Conflict of Interest

The authors declare that the research was conducted in the absence of any commercial or financial relationships that could be construed as a potential conflict of interest.
